# (5*S*,6*S*)-4,5-Dimethyl-3-methyl­acryloyl-6-phenyl-1,3,4-oxadiazinan-2-one

**DOI:** 10.1107/S1600536808013986

**Published:** 2008-05-17

**Authors:** Stanley A. Knott, Shawn R. Hitchcock, Gregory M. Ferrence

**Affiliations:** a CB 4160, Department of Chemistry, Illinois State University, Normal, IL 61790, USA

## Abstract

The title compound, C_15_H_18_N_2_O_3_, is an example of an oxadiazinan-2-one with significant inter­action between the N_3_-acyl and N_4_-methyl groups. These steric inter­actions result in a large torsion angle between the two carbonyl groups, not present with acyl substituents with less steric demand.

## Related literature

For related literature, see: Bruno *et al.* 2004 ▶; Burgeson *et al.* (2004 ▶); Casper *et al.* (2002*a* ▶,*b* ▶); Ferrence *et al.* (2003 ▶); Hitchcock *et al.* (2001 ▶, 2004 ▶); Szczepura *et al.* (2004 ▶); Trepanier *et al.* (1968 ▶).
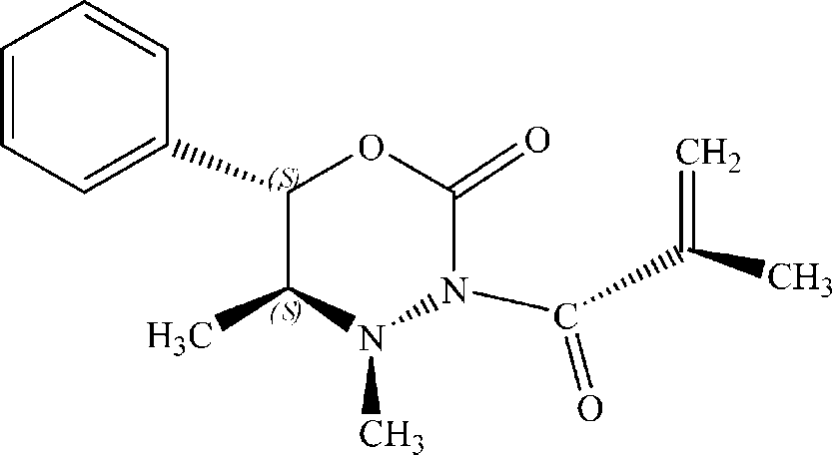


## Experimental

### 

#### Crystal data

C_15_H_18_N_2_O_3_
*M* *_r_* = 274.31
Orthorhombic, 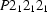

*a* = 8.7962 (6) Å
*b* = 9.7797 (6) Å
*c* = 16.6782 (11) Å
*V* = 1434.73 (16) Å^3^
*Z* = 4
Mo *K*α radiation
μ = 0.09 mm^−1^
*T* = 193 (2) K
0.46 × 0.38 × 0.21 mm

#### Data collection

Bruker P4/R4/SMART 1000 CCD diffractometer
Absorption correction: multi-scan (*SADABS* in *SAINT-Plus*; Bruker, 2003 ▶) *T*_min_ = 0.865, *T*_max_ = 0.982
9610 measured reflections
1702 independent reflections
1593 reflections with *I* > 2σ(*I*)
*R*_int_ = 0.032

#### Refinement

*R*[*F* ^2^ > 2σ(*F* ^2^)] = 0.032
*wR*(*F* ^2^) = 0.080
*S* = 1.07
1702 reflections
181 parameters
H-atom parameters constrained
Δρ_max_ = 0.15 e Å^−3^
Δρ_min_ = −0.13 e Å^−3^

### 

Data collection: *SMART* (Bruker, 2003 ▶); cell refinement: *SAINT-Plus* (Bruker, 2003 ▶); data reduction: *SAINT-Plus*; program(s) used to solve structure: *SIR2004* (Burla *et al.*, 2005 ▶); program(s) used to refine structure: *SHELXL97* (Sheldrick, 2008 ▶); molecular graphics: *ORTEP-3* for Windows (Farrugia, 1997 ▶); software used to prepare material for publication: *WinGX* (Farrugia, 1999 ▶) and *publCIF* (Westrip, 2008 ▶).

## Supplementary Material

Crystal structure: contains datablocks global, I. DOI: 10.1107/S1600536808013986/zl2116sup1.cif

Structure factors: contains datablocks I. DOI: 10.1107/S1600536808013986/zl2116Isup2.hkl

Additional supplementary materials:  crystallographic information; 3D view; checkCIF report

**Fig. 2.**
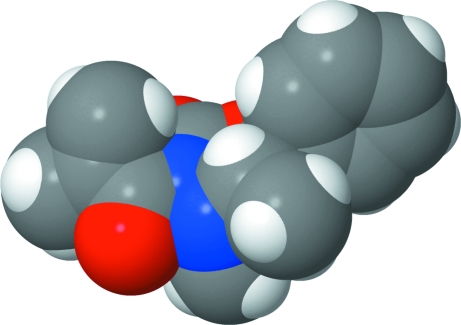
J mol enhanced figure of I. The default view shows the asymmetric unit which is the main residue depicted with a space-filling perspective. Several Jmol button scripts are provided to highlight key structural and crystallographic features.

Enhanced figure: interactive version of Fig. 2

## Figures and Tables

**Fig. 1.**
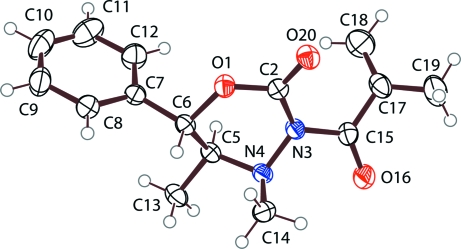
The molecular structure of compound (I), with the atomic numbering scheme. Displacement ellipsoids are drawn at the 50% probability level.

## supplementary crystallographic information

### Comment

In recent years, the synthesis of varying oxadiazinanones has led to chiral auxiliaries used in aldol addition reactions. The fundamental heterocyclic structure of related 1,3,4-oxadiazinan-2-one compounds has been known for nearly forty years (Trepanier *et al.*, 1968); however, it has not been until recent years that more detailed conformational studies have been performed on species containing an N_3_-acyl substituent (Hitchcock *et al.*, 2001; Casper *et al.*, 2002a). The influence of the N_3_-acyl substituent is of significant importance in these studies. When the N_3_-acyl substituent is of low steric demand the two carbonyls found in the molecule are co-planar and point at one another. However, when the N_3_-substituent is of high steric demand, repulsive steric interactions between the N_3_-acyl substituent and the N_4_-methyl group cause the two carbonyls to twist out of planarity. The molecule represented herein is an example of a structure in which the acyl substituent at the N_3_ position has a high steric demand.

Herein we report the single-crystal X-ray structure of a vinyl-acylated pseudoephedrine-derived 1,3,4-oxadiazinan-2-one (I). Several structures for various N_3_ substituted oxadiazananones have been published (Burgeson *et al.*, 2004; Casper *et al.*, 2002b; Ferrence *et al.*, 2003; Hitchcock *et al.*, 2001, 2004). Also, a compound unsubstituted at the N_3_ position has been synthesized (Szczepura *et al.*, 2004). A *Mogul* (Bruno *et al.* 2004) geometry check showed all non-H bond angles, distances and torsions to be within typical ranges. The structures of previously reported acetyl, propionyl, and *t*-butylacetyl substituents at the N_3_ position exhibit *syn*-parallel carbonyls. In contrast, in the title compound the 150.8 (2)° O16—C15—N3—C2 and 132.4 (3)° O20—C2—C15—O16 torsion angles are indicative of anti-parallel arrangement of the imide carbonyl groups. It appears likely that this arrangement helps to relieve steric congestion between the O16 carbonyl and the vinyl moiety while at the same time avoiding steric interactions between the N_4_ methyl group and the vinyl CH_2_ group. The O16—C15—C17—C18 torsion angle is 130.5 (2)°. The potential for steric interactions is further illustrated in the Jmol enhanced figure (Fig. 2).

### Experimental

The title compound was prepared by acylation of pseudoephedrine derived 1,3,4-oxadiazinan-2-one using sodium hexamethyldisilazane and methylacrylolyl chloride (Casper *et al.*, 2002a).

### Refinement

All non-H atoms were refined anisotropically without disorder, except for the C19 methyl group which had H-atoms attached as rotationally disordered methyl groups using the AFIX 123 command. All H atoms were initially identified through difference Fourier synthesis then removed and included in the refinement in the riding-model approximation (C–H = 0.95, 0.98, 0.99 and 1.00 Å for Ar–H, CH_3_ and *sp*^2^ CH_2_ and CH; *U*_iso_(H) = 1.2Ueq(C) except for methyl groups, where *U*_iso_(H) = 1.5Ueq(C)). In the absence of significant anomalous scattering effects, Friedel pairs were merged.

### Crystal data

**Table:**

C_15_H_18_N_2_O_3_	*F*_000_ = 584
*M**_r_* = 274.31	*D*_x_ = 1.27 Mg m^−^^3^
Orthorhombic, *P*2_1_2_1_2_1_	Mo *K*α radiation λ = 0.71073 Å
Hall symbol: P 2ac 2ab	Cell parameters from 7730 reflections
*a* = 8.7962 (6) Å	θ = 3.3–26.4º
*b* = 9.7797 (6) Å	µ = 0.09 mm^−^^1^
*c* = 16.6782 (11) Å	*T* = 193 (2) K
*V* = 1434.73 (16) Å^3^	Prism, colourless
*Z* = 4	0.46 × 0.38 × 0.21 mm

### Data collection

**Table:**

Bruker P4/R4/SMART 1000 CCD diffractometer	1702 independent reflections
Radiation source: sealed tube	1593 reflections with *I* > 2σ(*I*)
Monochromator: graphite	*R*_int_ = 0.032
*T* = 193(2) K	θ_max_ = 26.4º
/w scans	θ_min_ = 2.4º
Absorption correction: multi-scan(SADABS in SAINT-Plus; Bruker, 2003)	*h* = −10→11
*T*_min_ = 0.865, *T*_max_ = 0.982	*k* = −11→12
9610 measured reflections	*l* = −20→20

### Refinement

**Table:**

Refinement on *F*^2^	H-atom parameters constrained
Least-squares matrix: full	*w* = 1/[σ^2^(*F*_o_^2^) + (0.039*P*)^2^ + 0.2942*P*] where *P* = (*F*_o_^2^ + 2*F*_c_^2^)/3
*R*[*F*^2^ > 2σ(*F*^2^)] = 0.032	(Δ/σ)_max_ < 0.001
*wR*(*F*^2^) = 0.080	Δρ_max_ = 0.15 e Å^−^^3^
*S* = 1.07	Δρ_min_ = −0.12 e Å^−^^3^
1702 reflections	Extinction correction: none
181 parameters	

### Special details

**Table:**

Geometry. All e.s.d.'s (except the e.s.d. in the dihedral angle between two l.s. planes) are estimated using the full covariance matrix. The cell e.s.d.'s are taken into account individually in the estimation of e.s.d.'s in distances, angles and torsion angles; correlations between e.s.d.'s in cell parameters are only used when they are defined by crystal symmetry. An approximate (isotropic) treatment of cell e.s.d.'s is used for estimating e.s.d.'s involving l.s. planes.

### Fractional atomic coordinates and isotropic or equivalent isotropic displacement parameters (Å^2^)

**Table:**

	*x*	*y*	*z*	*U*_iso_*/*U*_eq_	Occ. (<1)
O1	0.56811 (14)	0.91142 (13)	0.46559 (7)	0.0296 (3)	
C2	0.6459 (2)	0.85642 (18)	0.40478 (10)	0.0255 (4)	
N3	0.56076 (17)	0.80251 (16)	0.34127 (8)	0.0270 (3)	
N4	0.40508 (18)	0.76608 (16)	0.35333 (9)	0.0281 (3)	
C5	0.3312 (2)	0.8846 (2)	0.39098 (11)	0.0275 (4)	
H5	0.3518	0.9668	0.357	0.033*	
C6	0.4005 (2)	0.90915 (19)	0.47323 (10)	0.0266 (4)	
H6	0.3726	0.8301	0.5083	0.032*	
C7	0.3531 (2)	1.0377 (2)	0.51526 (11)	0.0293 (4)	
C8	0.2870 (2)	1.0285 (2)	0.59025 (12)	0.0382 (5)	
H8	0.2767	0.9415	0.615	0.046*	
C9	0.2356 (3)	1.1443 (3)	0.63006 (14)	0.0501 (6)	
H9	0.1901	1.1364	0.6815	0.06*	
C10	0.2509 (3)	1.2699 (3)	0.59454 (16)	0.0548 (7)	
H10	0.2146	1.3495	0.6211	0.066*	
C11	0.3186 (3)	1.2809 (3)	0.52055 (17)	0.0581 (7)	
H11	0.3305	1.3685	0.4967	0.07*	
C12	0.3698 (3)	1.1662 (2)	0.48046 (14)	0.0432 (5)	
H12	0.4163	1.1749	0.4293	0.052*	
C13	0.1593 (2)	0.8641 (2)	0.39546 (12)	0.0368 (5)	
H13A	0.119	0.8483	0.3415	0.055*	
H13B	0.1119	0.9459	0.4184	0.055*	
H13C	0.1366	0.7849	0.4294	0.055*	
C14	0.3987 (2)	0.63742 (19)	0.39932 (11)	0.0345 (4)	
H14A	0.4509	0.5651	0.3694	0.052*	
H14B	0.2923	0.6114	0.4078	0.052*	
H14C	0.4486	0.6503	0.4513	0.052*	
C15	0.6276 (2)	0.7502 (2)	0.27061 (11)	0.0302 (4)	
O16	0.56288 (17)	0.66036 (17)	0.23419 (8)	0.0436 (4)	
C17	0.7710 (2)	0.8135 (3)	0.24081 (11)	0.0395 (5)	
C18	0.7866 (3)	0.9514 (3)	0.23698 (14)	0.0552 (7)	
H18A	0.8742	0.9903	0.2127	0.066*	
H18B	0.7099	1.009	0.2586	0.066*	
C19	0.8816 (3)	0.7179 (3)	0.20957 (19)	0.0697 (9)	
H19A	0.8464	0.6242	0.2191	0.105*	0.5
H19B	0.9793	0.7319	0.2366	0.105*	0.5
H19C	0.8941	0.7327	0.1518	0.105*	0.5
H19D	0.9668	0.7684	0.1859	0.105*	0.5
H19E	0.8339	0.6606	0.1684	0.105*	0.5
H19F	0.9191	0.6599	0.2532	0.105*	0.5
O20	0.78185 (15)	0.85606 (15)	0.40662 (8)	0.0334 (3)	

### Atomic displacement parameters (Å^2^)

**Table:**

	*U*^11^	*U*^22^	*U*^33^	*U*^12^	*U*^13^	*U*^23^
O1	0.0264 (7)	0.0361 (7)	0.0264 (6)	0.0026 (6)	−0.0026 (5)	−0.0042 (5)
C2	0.0276 (9)	0.0240 (9)	0.0249 (8)	0.0016 (7)	−0.0016 (7)	0.0032 (7)
N3	0.0232 (8)	0.0327 (8)	0.0250 (7)	−0.0007 (7)	0.0007 (6)	−0.0016 (6)
N4	0.0239 (8)	0.0330 (8)	0.0273 (7)	−0.0043 (7)	0.0012 (6)	−0.0013 (6)
C5	0.0253 (9)	0.0304 (10)	0.0267 (8)	−0.0014 (8)	0.0024 (7)	0.0034 (7)
C6	0.0255 (9)	0.0277 (9)	0.0266 (8)	−0.0010 (8)	0.0024 (8)	0.0026 (7)
C7	0.0252 (9)	0.0312 (10)	0.0315 (9)	−0.0004 (8)	−0.0011 (8)	−0.0018 (8)
C8	0.0376 (11)	0.0409 (12)	0.0360 (10)	−0.0041 (10)	0.0027 (9)	−0.0055 (9)
C9	0.0417 (12)	0.0642 (16)	0.0445 (11)	0.0043 (12)	0.0035 (10)	−0.0207 (12)
C10	0.0533 (15)	0.0482 (14)	0.0628 (15)	0.0179 (12)	−0.0107 (13)	−0.0232 (12)
C11	0.0741 (18)	0.0294 (12)	0.0707 (17)	0.0106 (12)	−0.0103 (15)	−0.0008 (12)
C12	0.0514 (13)	0.0342 (11)	0.0442 (11)	0.0028 (10)	0.0044 (10)	0.0044 (10)
C13	0.0248 (9)	0.0471 (12)	0.0384 (10)	−0.0031 (9)	0.0016 (8)	0.0015 (10)
C14	0.0386 (10)	0.0299 (10)	0.0349 (9)	−0.0052 (9)	0.0028 (9)	−0.0033 (8)
C15	0.0278 (10)	0.0379 (11)	0.0249 (8)	0.0029 (8)	−0.0018 (7)	−0.0026 (8)
O16	0.0390 (8)	0.0533 (9)	0.0385 (7)	−0.0053 (8)	0.0051 (6)	−0.0169 (7)
C17	0.0302 (10)	0.0612 (14)	0.0272 (9)	−0.0053 (10)	0.0019 (8)	−0.0060 (9)
C18	0.0665 (17)	0.0539 (15)	0.0453 (12)	−0.0102 (14)	0.0131 (12)	0.0109 (11)
C19	0.0401 (14)	0.089 (2)	0.0800 (18)	−0.0012 (15)	0.0159 (14)	−0.0317 (17)
O20	0.0253 (7)	0.0426 (8)	0.0324 (6)	0.0028 (6)	−0.0047 (5)	−0.0008 (6)

### Geometric parameters (Å, °)

**Table:**

O1—C2	1.336 (2)	C11—C12	1.382 (3)
O1—C6	1.480 (2)	C11—H11	0.95
C2—O20	1.196 (2)	C12—H12	0.95
C2—N3	1.400 (2)	C13—H13A	0.98
N3—C15	1.413 (2)	C13—H13B	0.98
N3—N4	1.429 (2)	C13—H13C	0.98
N4—C5	1.470 (2)	C14—H14A	0.98
N4—C14	1.475 (2)	C14—H14B	0.98
C5—C6	1.520 (2)	C14—H14C	0.98
C5—C13	1.528 (3)	C15—O16	1.210 (2)
C5—H5	1	C15—C17	1.491 (3)
C6—C7	1.499 (3)	C17—C18	1.357 (4)
C6—H6	1	C17—C19	1.447 (3)
C7—C8	1.382 (3)	C18—H18A	0.95
C7—C12	1.392 (3)	C18—H18B	0.95
C8—C9	1.388 (3)	C19—H19A	0.98
C8—H8	0.95	C19—H19B	0.98
C9—C10	1.371 (4)	C19—H19C	0.98
C9—H9	0.95	C19—H19D	0.98
C10—C11	1.375 (4)	C19—H19E	0.98
C10—H10	0.95	C19—H19F	0.98
			
C2—O1—C6	124.71 (14)	C5—C13—H13B	109.5
O20—C2—O1	119.58 (17)	H13A—C13—H13B	109.5
O20—C2—N3	123.57 (17)	C5—C13—H13C	109.5
O1—C2—N3	116.85 (15)	H13A—C13—H13C	109.5
C2—N3—C15	123.02 (15)	H13B—C13—H13C	109.5
C2—N3—N4	119.99 (14)	N4—C14—H14A	109.5
C15—N3—N4	115.19 (14)	N4—C14—H14B	109.5
N3—N4—C5	106.68 (14)	H14A—C14—H14B	109.5
N3—N4—C14	108.80 (14)	N4—C14—H14C	109.5
C5—N4—C14	115.71 (14)	H14A—C14—H14C	109.5
N4—C5—C6	109.45 (14)	H14B—C14—H14C	109.5
N4—C5—C13	110.80 (16)	O16—C15—N3	119.08 (18)
C6—C5—C13	111.93 (15)	O16—C15—C17	122.15 (18)
N4—C5—H5	108.2	N3—C15—C17	118.67 (17)
C6—C5—H5	108.2	C18—C17—C19	123.9 (3)
C13—C5—H5	108.2	C18—C17—C15	120.9 (2)
O1—C6—C7	107.77 (14)	C19—C17—C15	114.9 (2)
O1—C6—C5	108.91 (14)	C17—C18—H18A	120
C7—C6—C5	116.28 (15)	C17—C18—H18B	120
O1—C6—H6	107.9	H18A—C18—H18B	120
C7—C6—H6	107.9	C17—C19—H19A	109.5
C5—C6—H6	107.9	C17—C19—H19B	109.5
C8—C7—C12	118.74 (19)	H19A—C19—H19B	109.5
C8—C7—C6	119.06 (18)	C17—C19—H19C	109.5
C12—C7—C6	122.18 (17)	H19A—C19—H19C	109.5
C7—C8—C9	121.1 (2)	H19B—C19—H19C	109.5
C7—C8—H8	119.4	C17—C19—H19D	109.5
C9—C8—H8	119.4	H19A—C19—H19D	141.1
C10—C9—C8	119.5 (2)	H19B—C19—H19D	56.3
C10—C9—H9	120.3	H19C—C19—H19D	56.3
C8—C9—H9	120.3	C17—C19—H19E	109.5
C9—C10—C11	120.0 (2)	H19A—C19—H19E	56.3
C9—C10—H10	120	H19B—C19—H19E	141.1
C11—C10—H10	120	H19C—C19—H19E	56.3
C10—C11—C12	120.8 (2)	H19D—C19—H19E	109.5
C10—C11—H11	119.6	C17—C19—H19F	109.5
C12—C11—H11	119.6	H19A—C19—H19F	56.3
C11—C12—C7	119.8 (2)	H19B—C19—H19F	56.3
C11—C12—H12	120.1	H19C—C19—H19F	141.1
C7—C12—H12	120.1	H19D—C19—H19F	109.5
C5—C13—H13A	109.5	H19E—C19—H19F	109.5
			
C6—O1—C2—O20	−175.30 (16)	C5—C6—C7—C8	122.65 (19)
C6—O1—C2—N3	5.1 (3)	O1—C6—C7—C12	66.8 (2)
O20—C2—N3—C15	−4.2 (3)	C5—C6—C7—C12	−55.7 (2)
O1—C2—N3—C15	175.31 (16)	C12—C7—C8—C9	1.1 (3)
O20—C2—N3—N4	159.76 (17)	C6—C7—C8—C9	−177.33 (19)
O1—C2—N3—N4	−20.7 (2)	C7—C8—C9—C10	−0.2 (3)
C2—N3—N4—C5	50.5 (2)	C8—C9—C10—C11	−0.9 (4)
C15—N3—N4—C5	−144.27 (15)	C9—C10—C11—C12	1.1 (4)
C2—N3—N4—C14	−74.94 (19)	C10—C11—C12—C7	−0.2 (4)
C15—N3—N4—C14	90.25 (17)	C8—C7—C12—C11	−0.9 (3)
N3—N4—C5—C6	−64.27 (17)	C6—C7—C12—C11	177.5 (2)
C14—N4—C5—C6	56.91 (19)	C2—N3—C15—O16	150.77 (18)
N3—N4—C5—C13	171.82 (15)	N4—N3—C15—O16	−13.9 (3)
C14—N4—C5—C13	−67.0 (2)	C2—N3—C15—C17	−32.8 (3)
C2—O1—C6—C7	−147.65 (16)	N4—N3—C15—C17	162.50 (16)
C2—O1—C6—C5	−20.7 (2)	O16—C15—C17—C18	130.5 (2)
N4—C5—C6—O1	50.02 (18)	N3—C15—C17—C18	−45.8 (3)
C13—C5—C6—O1	173.25 (16)	O16—C15—C17—C19	−43.4 (3)
N4—C5—C6—C7	171.95 (15)	N3—C15—C17—C19	140.3 (2)
C13—C5—C6—C7	−64.8 (2)	O20—C2—C15—O16	132.4 (3)
O1—C6—C7—C8	−114.81 (19)		
